# Alternative consent methods used in the multinational, pragmatic, randomised clinical trial SafeBoosC-III

**DOI:** 10.1186/s13063-024-08074-0

**Published:** 2024-04-04

**Authors:** Maria Linander Vestager, Mathias Lühr Hansen, Gorm Greisen, the SafeBoosC-III trial group, Adelina Pellicer, Caitriona Ni Chathasaigh, Chantal Lecart, Claudia Knoepfli, Cornelia Hagmann, Dario Gallo, Ebru Ergenekon, Eleftheria Hatzidaki, Eugene Dempsey, Evangelina Papathoma, Gabriel Dimitrou, Gerhard Pichler, Gitte Holst Hahn, Gunnar Naulaers, Hans Fuchs, Hilal Ozkan, Isabel de las Cuevas, Itziar Serrano-Viñuales, Jan Sirc, Julie de Buyst, Kosmos Sarafidis, Luis Arrusa, Mariana Baserga, Martin Stocker, Merih Cetinkaya, Miguel Alsina, Monica Fumagalli, Olalla Otero Vaccarello, Olivier Baud, Pamela Zafra-Rodríguez, Pierre Maton, Quoqiang Cheng, Ruth del Rio Florentino, Ryszard Lauterbach, Salvador Piris-Borregas, Saudamini Nesargi, Siv Fredly, Sylwia Marciniak, Tomasz Szczapa, Xiaoyang Gao, Xin Xu

**Affiliations:** 1 Department of Neonatology, Copenhagen University Hospital – Rigshospitalet, Copenhagen, Denmark; 2 Copenhagen Trial Unit, Centre for Clinical Intervention Research, The Capital Region, Copenhagen University Hospital – Rigshospitalet, Copenhagen, Denmark

**Keywords:** Neonatal, Consent, Ethic, Trial, Prior consent, Deferred consent, Opt-out consent

## Abstract

**Background:**

The process of obtaining prior informed consent for experimental treatment does not fit well into the clinical reality of acute and intensive care. The therapeutic window of interventions is often short, which may reduce the validity of the consent and the rate of enrolled participants, to delay trial completion and reduce the external validity of the results. Deferred consent and ‘opt-out’ are alternative consent methods. The SafeBoosC-III trial was a randomised clinical trial investigating the benefits and harms of cerebral oximetry monitoring in extremely preterm infants during the first 3 days after birth, starting within the first 6 h after birth. Prior, deferred and opt-out consent were all allowed by protocol.

This study aimed to evaluate the use of different consent methods in the SafeBoosC-III trial, Furthermore, we aimed to describe and analyse concerns or complaints that arose during the first 6 months of trial conduct.

**Methods:**

All 70 principal investigators were invited to join this descriptive ancillary study. Each principal investigator received a questionnaire on the use of consent methods in their centre during the SafeBoosC-III trial, including the possibility to describe any concerns related to the consent methods used during the first 6 months of the trial, as raised by the parents or the clinical staff.

**Results:**

Data from 61 centres were available. In 43 centres, only prior informed consent was used: in seven, only deferred consent. No centres used the opt-out method only, but five centres used prior and deferred, five used prior, deferred and opt-out (all possibilities) and one used both deferred and opt-out. Six centres applied to use the opt-out method by their local research ethics committee but were denied using it. One centre applied to use deferred consent but was denied. There were only 23 registered concerns during the execution of the trial.

**Conclusions:**

Consent by opt-out was allowed by the protocol in this multinational trial but only a few investigators opted for it and some research ethics boards did not accept its use. It is likely to need promotion by the clinical research community to unfold its potential.

**Supplementary Information:**

The online version contains supplementary material available at 10.1186/s13063-024-08074-0.

## Introduction

The use of prior informed consent is the standard in clinical trials and must comply with the requirements stated in The Declaration of Helsinki [1]. In neonatal or paediatric research, the parents or legal guardians must give informed consent [2, 3]. To improve neonatal care, randomised clinical trials are needed. Unfortunately, the process of obtaining prior informed consent for experimental treatment does not fit well into the clinical reality of acute and intensive care. The intervention often has a short therapeutic window, thus making it difficult to obtain valid prior informed consent in time. This problem may seriously reduce the value of the consent and/or the number of enrolled children and thereby inhibit trial execution and completion. Other consent methods may therefore be more appropriate for intensive care research. Deferred consent is commonly accepted for acute illness in adults who are unable to consent themselves at the time of randomisation, where in that scenario, a family member will be asked to provide assent. An alternative is prior informed assent (opt-out), which does give some information and does allow prospective participants an opportunity to decline participation [4].

Multiple studies have examined the ethical aspects of obtaining consent in an acute setting. Many aspects and groups of involved parts—practitioners (doctors, nurses) and families (parents, grandparents)—make the interpretation and discussion multifaceted. In an observational study based on the opinions of parents of newborns enrolled into a neonatal research study, 89% of the parents (*n* = 100) were ‘satisfied’ with the deferred consent process [5]. A qualitative study examining the use of opt-out in a neonatal randomised trial across eleven neonatal units in England, found the opt-out method to be feasible and acceptable by health professionals. In addition, parents did not see opt-out consent as undermining their right to decide for their child [6]. Regardless of this, both studies describe difficulties with implementation of other consent methods than prior informed consent.

Our study aimed to evaluate the use of different consent methods in the SafeBoosC-III trial, based on what was chosen by the local investigators and what was accepted by the Research Ethics Boards (REBs) across different countries. Furthermore, we aimed to describe and analyse concerns or complaints that arose during the first 6 months of trial conduct from both practitioners and families.

## Methods/design

This descriptive ancillary study is based on the SafeBoosC-III trial [7], which was a randomised clinical trial investigating the benefits and harms of treatment based on cerebral near-infrared spectroscopy. The hypothesis was that treatment based on cerebral oximetry monitoring during the first 72 h of life of extremely preterm infants would result in a reduction of severe brain injury and death at 36 weeks postmenstrual age [7]. In December 2021, recruitment was completed with a total of 1601 extremely preterm newborns. The trial took place in 70 centres across 17 countries. The trial was of pragmatic design, meaning it should be of as little disturbance in the clinical routines as possible. Trial enrollment was done by the attending clinical staff, who were also responsible for the clinical care of trial participants, and therefore consent must be fast and simple. Furthermore, legal requirements differ among countries and therefore, different consent methods were allowed by the trial protocol. To support the principal investigators' decision about which consent method to apply for, the trial protocol included an appendix with pros and cons regarding the three different, allowed consent methods, as described under the ‘Introduction’ section [8].

In deferred consent, informed consent is sought after enrolling the patients into a trial and if not given, any intervention will be withdrawn, and no data will be used. Since it was first described in the literature in 1980 [9] it is now accepted by regulation in the European Union [10], but only if certain criteria are met. Studies have evaluated doctors' views on deferred consent and the general tendency was that previous experience with deferred consent resulted in a positive approach and vice versa [11].

In the opt-out method, enrollment happens as default. The parents will be informed before the intervention (as is the standard for clinical care) of ongoing research, and it should be recorded in the clinical file, that this information has been given. The parents can opt, out and—since they are informed—have the possibility, and right to withdraw their consent at any time [12].

To increase the value of the SafeBoosC-III trial, this study was designed as a study-within-a-trial (SWAT) [13]. The principal investigator of all centres participating in the SafeBoosC-III trial received an invitation to participate in this ancillary study, prior to having obtained ethics approval. The invitation also included a questionnaire with six close-ended questions and one open-ended question with free-text answers. The questions were:

- Date of protocol submission to research ethics board (date)
- Date of final decision by research ethics board (date)
- What consent method did your neonatal intensive care unit apply to use (prior informed consent, deferred consent, opt-out)?
- Did the research ethics board raise any queries regarding this consent method (yes/no)?
- If the research ethics board raised any queries towards the consent procedure, please specify (free text answer)?
- Did the research ethics board grant final approval of this consent method (yes/no)?
- What consent method(s) did you end up using in the trial (prior informed consent, deferred consent, opt-out)?

The questionnaire also included a section which requested the principal investigator to register, describe and report any concerns or complaints in relation to the consent procedure, as raised by clinical staff or parents of the trial participants during the first 6 months of trial conduct in their centre.

As we did not have any specific hypothesis, no tests of statistical significance were made, and the analysis of quantitative data was limited to simple counts and visionalisation. Due to the small number of comments, only a simple grouping of the qualitative data was made.

### Outcomes

The primary outcome of this study was the number of participating centres using prior informed consent, deferred consent and opt-out.

Secondary outcomes were:

- The number of investigators choosing to apply for the use of either prior, opt-out or deferred informed consent.
- The number of applications for ‘opt-out’ and deferred informed consent that was not granted by the research ethics board.
- The number of research ethics boards raising queries towards the consent method; and
- Time to decision from submission of the application.

The exploratory outcomes were:

- Research ethics boards queries towards the consent method; and
- Concerns or complaints concerning the consent method raised by clinical staff or parents during the first 6 months of the trial.

## Results

A total of 62 centres out of 70 (88%) accepted to participate and 61 centres (87%) ended up delivering the data.

Eleven centres (18% of the centres delivering the data) used more than one method of consent: five centres (8%) used all three (prior, deferred and opt-out), five (8%) used prior and deferred and one (2%) used deferred and opt-out. Fifty centres (82%) used only one method of consent: 43 (70%) used prior informed consent, seven (11%) deferred consent, and no centre used only opt-out.

**Fig. 1:**
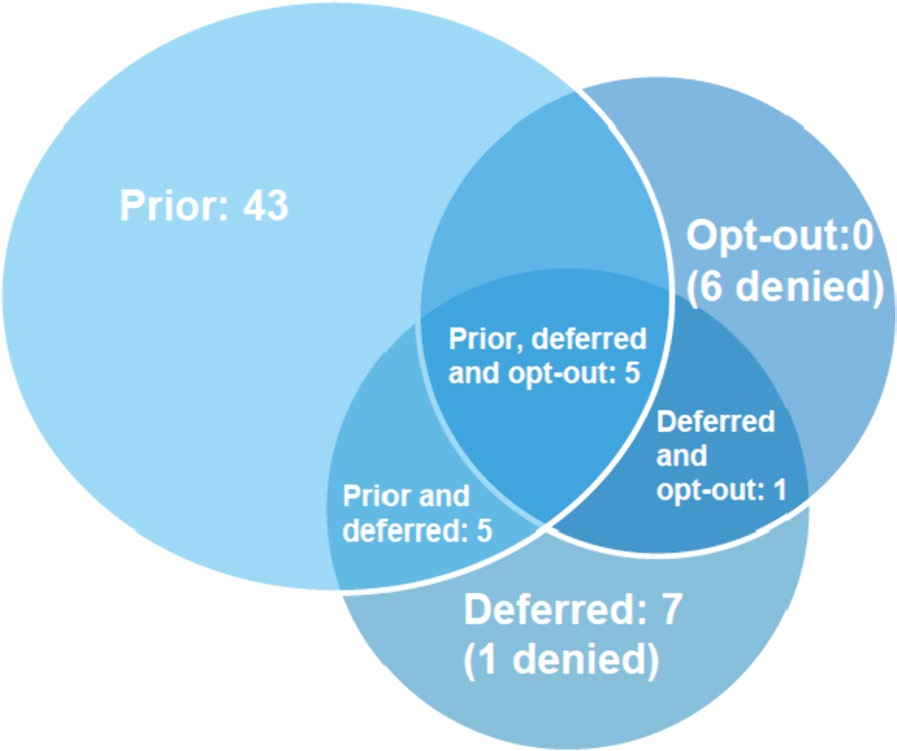
The consent methods used in the SafeBoosC-III trial, by centre

Six centres (10%) were declined the use of opt-out by the research ethics board, and one (2%) was declined the use of deferred consent (Fig. 1).

**Fig. 2:**
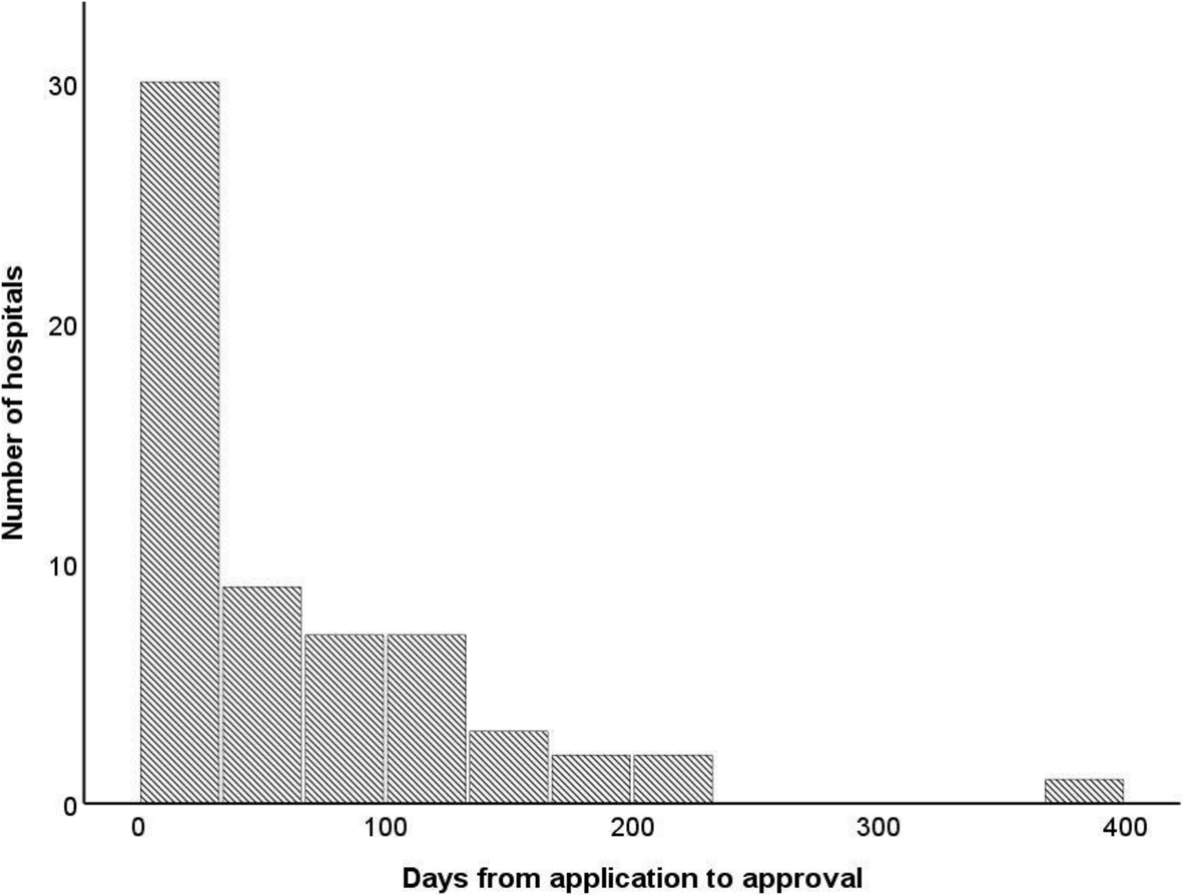
Days from application to approval

The median time to decision from submission of the application was 34 days (Fig. 2) (inter-quartile range 75 (15–90)).

### Concerns

The REB queries towards the consent method (*n* = 6) are listed in Table 1. In four out of six cases, the consent method applied was ultimately accepted. In one case, the use of deferred consent was declined. Five out of six cases were from Europe and the last from the USA.

**Table 1:** The REB queries towards the consent method

What consent method did your hospital apply to use?	If the REB raised any queries towards the consent procedure, please specify:	Did the REB grant final approval for this consent method?
*Denmark*: deferred consent	The REB did not find that the time limit of 6 h and the fact that the mother is always in the hospital could justify deferred consent. Opt-out is not legal.	No
*Greece*: prior, deferred and opt-out	We applied for prior informed consent (standard or ‘opt-out’, depending on the situations surrounding preterm birth, such as time of birth and parental psychological status) and we also asked approval for deferred consent on very specific occasions (e.g. transfer of the neonate from another hospital and single parent family with the mother presenting serious complications *postpartum*). Our Ethics Committee approved only the **prior informed consent (standard or ‘opt-out’**, as analysed above). Their rationale was that SafeboosC is not just an observational study but involves interventions which might change the course of the disease. Even if such changes would be for the patient’s short- or long-term benefit, the members of the committee argued that the parents should be informed and give their consent in advance.	Approved only the prior informed consent (standard or ‘opt-out’)
*Spain*: prior, deferred and opt-out	They wanted a further explanation on the opt-out and deferred consent. To be acceptable for the members that an infant is randomised without prior consent, I should make a statement with regards to ‘being in the control group would not be a prejudice, because the standard treatment would be adopted; and being in the intervention group would potentially have benefits’ With this arguments, they were happy and the study was approved. They also wanted me to know about the discussion they had when SafeBoosC-III was presented at REB, as the President considered that this kind of trial should have a waiver for parental consent, as could be considered routine practice.	Yes
*Spain*: prior, deferred and opt-out	The REB included a suggestion in its report to insist that parents must consent.	Yes
*Switzerland*: prior and deferred	In Switzerland no opt-out for clinical trials. Clarify the declaration of intent and signature of an independent doctor. We decided to resubmit with the following process and justification: Contact the parents before inclusion whenever possible and get informed consent. If not possible and if there are no obvious signs against an inclusion, randomisation might be done and monitoring started. However, informed consent must be obtained as fast as possible. This option allows parents to think about it without missing study participation.	Yes
*USA*: prior	IRB requires consent from only one parent or legal guardian IRB required that future use of collected data must be made optional with the choice to opt out	Yes

The concerns reported during the trial were of two main types: concerns about information and consent, e.g. the time constraints in the clinical context, parental competence and reflections on the various options for obtaining consent (*n* = 13) (Table 2) and concerns about the trial as such, e.g. risks of harm by NIRS or threats to privacy (*n* = 9) (Table 3). In 15 out of 22 (68%) cases, the concern was made by staff (consultant, resident, nurse of staff member). The remaining concerns (32%) were made by family. In Table 2, part A: ‘ethical concerns regarding information and consent’, there was a consequence or solution of the concern in 71% of the cases and in part B: ‘concerns regarding the consent procedures’ a solution was found in 4 of 6 (67%). For ‘Questions or concerns regarding the trial as such’, a solution was found in 6 of 9 (67%) (Table 3).

**Table 2:** Concerns raised by clinical staff or parents during the first 6 months of the trial

Who raised the concern?	Subject	Consequence or solution
**Part A:** Ethical concerns regarding information and consent		
*Consultant*	Some find it uncomfortable to bother the parents with the study consent so soon after delivery.	We are trying, whenever possible, to include the patient before delivery
*Consultant*	How much time after birth is it ethical to obtain deferred consent?	We agreed on ‘as soon as possible’, balancing the family´s emotional situation but also fairness not to limit their right to consent. or not to participate in the study.
*Consultant*	There is uncertainty about when the baby will be born. Asking for prenatal consent is perceived as an added stressor for the parents.	The opt-out model is perceived as the best prenatal option, an attempt will be made to implement
*Consultant*	Wonder if the father of an ELBW (extremely low birth weight infant) in critical care, even after correct information, is in an appropriate emotional state to make such a decision.	None
*Consultant*	It is difficult to talk about a baby who is even not born. He/she may need resuscitation and may also die. Also, parents can easily get confused and don’t understand the things that give information about the prognosis of a baby.	None
*Parents*	Most parents were anxious about the survival and prognosis of their babies, they were afraid for their baby as they thought of the procedure as an experiment rather than a clinical study.	After a detailed explanation, most of them agreed to give consent prior to birth.
*Resident*	Is it ethical to use deferred consent?	Deferred consent and ethical issues were discussed with medical staff. Resident was satisfied and practice was not changed
**Part B:** Concerns regarding the consent procedures		
*Consultant*	Limited time to obtain signed informed consent before randomisation. Risk of reduced patient recruitment.	None
*Consultant*	It has been difficult to incorporate the opt-out model due to not having a good understanding of the procedure.	It will probably be used more from now on after clarifying doubts in the monitoring visit. The opt-out model will be used in prenatal visits.
*Consultant*	The postnatal model is complicated by the large number of tasks in the first hours of life.	Deferred models are preferably used.
*Consultant*	Short time period for those who presented in labour and did not have time for an antenatal consent process.	None
*Consultant*	When the delivery is urgent and the Obgyn takes the mother to delivery, it becomes impossible to get a prior informed consent and these babies are excluded from the study.	Deferred consent may be helpful in these urgent cases
*Parents*	The patient was allocated in the control group and was born during a long weekend, so the doctor on duty gave the parents the consent form in the NICU with basic information about the study. They initially rejected the study arguing that they had been asked prenatally too many times to participate in studies. Five days later, I asked them to talk about the study outside the NICU and I explained in detail the study purpose and what participating in the control group meant. Finally, they accepted the study and they told me that they had changed their minds thanks to the information.	I think that for doctors on duty sometimes is difficult to explain the study to the parents with detail an in a comfortable environment. However, especially for patients in the experimental group, it is necessary to ask them as soon as possible, although we are using deferred consent.

**Table 3:** Questions or concerns regarding the trial as such

Who raised the concern?	Subject	Consequence or solution
*Consultant*	In case of not be allowed to include the baby in the trial, are we allowed to use the data collected until that time?	None
*Family*	What will happen if there is no agreement in the trial participation between two parents?	We decided that participation will be only considered when two parents agree in the participation. We included this issue in the verbal information to the families.
*Grandparent*	Concern about the safety of NIRS.	Give the parents a detailed interpretation about the NIRS monitoring
*Mother*	In case of giving permission to use the data of my baby, the data used for the study could identify my baby?	We gave a detailed explanation regarding the protection of personal data. We explained to the parents that the personal data of the baby won’t be included in the database. Only clinical data will be used.
*Nurse*	Nurse’s point of view was that the explanation in the CI was extremely technical. In their opinion, it was too technical and it needed to be explained more simplified with more algorithms.	We modified the CI by adding more pictures explaining NIRS and also explaining the timing. Moreover, we made a PowerPoint presentation which was available in all the computers for all the staff to check all the information about the study.
*Parent*	Will it harm the baby?	None
*Parent*	Is there a cost involved for the use of the machine?	None
*Staff member*	Will be any kind of data used if the consent form was declined by the family?	We asked to the principal investigator when this query was raised. They confirmed to us that any kind of data will be used when the family decline the participation in the trial.
*Resident*	About clinical staff, the concern was the time of randomisation. Some preterm infants admitted to the hospital beyond 6 h of birth had no chance to take part in the trial.	It is a bit difficult for us to control it.

## Discussion

Prior informed consent was most used. In some cases, the use of deferred consent or opt-out was declined by research ethics boards. In the centres using deferred consent and opt-out, there were few complaints concerning the ethical aspect of the consent method from staff or parents.

### The ethical difficulties for clinical trials testing interventions shortly after birth

In practice, there is wide variation in the situation of the parents who are the legal representatives and who may give surrogate consent.

In some cases, delivery is expected for days or weeks, and there is ample time to inform, and parents may be at their full capacity to provide consent. On the other hand, it may be considered inappropriate to take consent at that time, due to the fact that the situation is still hypothetical, Furthermore, if threatened preterm delivery is postponed, then trials that have an upper limit to the gestational age at birth, such as the SafeBoosC-III trial, may not be able to enrol all infants of parents who were informed and did consent.

In other cases, delivery is unprepared and catastrophic. The mother may be affected by complications of pregnancy, delivery and/or anaesthesia and the father affected by concerns for the life or health of the mother as well as the child. Even after a well-prepared preterm delivery, parents may be visibly affected by distress, to affect their level of competence, making information and consent shortly after delivery potentially further distressing and of questionable validity.

And yet, other parents, the mother, the father, or both, may stay calm, realistic and appear to be able to take in new information and make rational, personal choices.

In these troubled waters, selection biases are induced by the judgement of the staff on whether it is appropriate to approach the parents for consent (as several of the reported ‘concerns’ in this study demonstrate) as well as by the judgement of parents (in their balancing the understanding they can master in the situation and the trust they have with their perceptions of benefits and risks of the trial and the obligations they feel to contribute to research). Typically, infants that are enrolled in clinical trials shortly after birth that require parental consent are ‘healthier’ and their parents of better education than those who are not enrolled [14]. Such biases may reduce the generalizability of the results of these trials.

Given this variation and the multinational organisation of the SafeBoosC-III trial, the protocol allowed the use of prior or deferred consent or opt-out as decided by the local primary investigator and approved by the local REB.

### The REB decision time

As expected, we saw a wide range of opinions and processing time from different REBs. In one-quarter of the centres, the processing time exceeded 90 days.

With a processing time of close to a year in some countries, the research pathway will be slowed down. Relevant questions are if this is due to over-loaded research ethics committees, incomplete applications, ineffective bureaucratic processes, or due to fair deliberations of the pros and cons of the ethics of the trial as such and the alternative information and consent processes by the REB with representation of the relevant parties.

Many of the centres in the SafeBoosC-III trial were European. In the EU legislation, it is pointed out that *the timelines for assessing an application should be sufficient to assess the file while, at the same time, ensuring quick access to new, innovative treatments and ensuring that the Union remains an attractive place for conducting clinical trials* [10]*.* This supports the idea of more collaboration between international REBs and more knowledge-sharing on research ethics and methods, including informed consent, which for now is considered an aspect of national affairs. In most European countries research involving humans is reviewed independently by multidisciplinary research ethics committees, but the legislation regarding consent varies even among similar countries (e.g. Denmark, Finland and Norway) [15].

### REB decline of deferred consent or opt-out

This happened in about 30% of the centres where it was applied for. We do not have details about the deliberations, but the small number of applications shows that most primary investigators choose to apply for prior informed consent in the first place.

### Concerns and complaints

The SafeBoosC-III trial enrolled 1601 infants in 70 centres over 30 months. Thus, a report of only 23 concerns overall may be interpreted as a small number. The reported concerns focus on the timing of information and consent. The SafeBoosC-III trial enrolled extremely preterm infants and the intervention (monitoring of cerebral oxygenation by putting a near-infrared sensor on the head of the infant) had to be started within 6 h after birth. Thus, it is no surprise it is a delicate matter. Balancing the ethical tension with the need to include patients in a certain timeframe represents an ongoing challenge. There were concerns regarding the implementation of deferred consent and ‘opt-out’, but this appeared to be due to inexperienced investigators, rather than due to concerns with the principles. This is in line with previous studies [11].

### Unsuccessful deferred consent

Using five criteria for acceptability of clinical trials of urgent treatment of patients who are unable to give prior consent, it can be argued that deferred consent was appropriate for the SafeBoosC-III trial: (a) the intervention under test responded to an urgent medical need, (b) it had an a priori favourable benefit-risk balance, (c) it did not involve aspects which would be against the values or preferences of the infant, (d) it had little net risk, (e) consent would be obtained as soon as reasonably possible [16].

A total of 504 infants (31%) were enrolled in the trial by deferred consent, but for 18 (4%) of them ultimately parents did not give consent [7]. This represents a double problem: first, the fact that parents have to face that their child was enrolled in research they did not approve of represents an ethical problem, second, as the parents did not authorise the use of their child’s data, it constitutes a loss to follow-up, which is a methodological problem. This seems to be an inherent risk with this method of consent. For comparison, for only three of 1082 (0.3%) infants, the parents withdrew consent given before enrollment. In Denmark, deferred consent was declined by the REB, since the parents cannot generally be said to be incompetent even immediately after the birth of an extremely preterm infant.

### The rare use of opt-out

Only 16 infants (1%) were enrolled after opt-out, which is similar to a ‘clinical-routine’ method of consent. Brief information including the fact that the infants will be enrolled in the trial is provided and parents are given the chance to ask for more information or to opt-out. Their opt-out will be respected and no reasons will be asked for. In the clinical situation, in contrast, if parents decline a treatment that the physician believes is needed, there may be pressure, and potentially also means to challenge the parental custody and treat the infant against the will of the parents.

If parents do not respond, the infant will be enrolled. This way of presenting the choice may be considered as a form of nudging, drawing on what is known as the ‘status quo bias’ [17]. Also, since there is no signed consent form, but only a note on the consent process by the clinician-investigator in the clinical records, there is no evidence that the parents ever understood that they had a choice. On the other hand, it has been documented that even after a conventional process of prior information and consent for urgent neonatal trials, parents may report problems with information, understanding, competence as well as voluntariness [18].

In this light, opt-out may be seen as fair, i.e. the responsibility for enrolling the infant remains clearly with the investigator and seen as rationally appropriate for ‘comparative effectiveness research’ when the clinician-investigator is in equipoise as regards the benefits and risk of the alternative treatment options [4]. In practice, it may be difficult for clinician-investigators not to apply it as a ‘verbal opt-in’ method [6]. Several of the reported ‘concerns’ illustrate the need for education and training to implement opt-out properly.

Compared to deferred consent or waiver of consent, the opt-out method also implements the ethical value of transparency, which relies on honesty, respect and responsibility. Our interpretation is that opt-out should be used more often, especially in comparative effectiveness research as in the SafeBoosC-III trial, which we view and have described as a pragmatic trial with minimal risk. In the best-case scenario, the intervention will benefit the intervention group. The use of cerebral oximetry is already in clinical use across the globe. An international survey questionnaire from 2018 showed that 85 out of 235 neonatal intensive care units owned a cerebral oximeter and 69 used it for clinical purposes [19]. As for adults [20], a Cochrane review has previously stated that the evidence to recommend the implementation of cerebral oximetry in intensive care of newborns is not available [21]. A recent meta-analysis including 23 randomised controlled trials in all types of patients [22] concluded there is a need for another 3000 participants to draw a conclusion. Due to the lack of sponsorship from device manufacturers, compared to the role of the pharmaceutical industry in drug trials, this can only realistically be obtained by pragmatic trials where informed consent can be integrated into clinical care.

Another context where obtaining consent from a family in distress is relevant, is for non-therapeutic research, i.e. when there is no direct benefit for a child to enter a trial, e.g. drawing a blood sample. Here, the focus is not on the autonomy of parents but on the risk to the child. In most European countries it is only legal if it involves no more than minimal risk [15].

### Strengths and weaknesses

The study protocol was available from the start, including the templates for reporting the approval process with the REB and for the report of complaints by staff, parents or family. Nine of the 70 principal investigators representing the participating centres choose not to take part in this ancillary study. No reminders were sent, and it is not known to which extent it was made clear to everybody involved during the trial that this ancillary study existed.

The heterogeneity of the jurisdiction in the participating countries represents a strength and a weakness in this study. On one side, the results are representative of international research ethics as expressed by the choice of consent methods applied for by primary investigators, approved by REBs and perceived by staff and parents. On the other side, some options are legally impossible in given countries, e.g. the opt-out method is not allowed in Denmark and Switzerland.

### What did this study add?

This study supports a previous study by Woolfall et al. [11] showing that lack of experience with other methods of consent than standard prior informed consent may contribute to their under-utilisation. In that study, a barrier was found in the practitioners, even though all had recruited participants to trials before (*n* = 45). Woolfall et al., however, are focused on deferred consent and also address the potential compromising of autonomy by this method. Further, in contrast to our study, parents were not asked for their views on the consent process.

### What’s next?

In our opinion, opt-out has a rational place in comparative effectiveness research—where two clinical practices that are already in common use are compared. Principally, it is ‘better’ than deferred consent as the parents do get a say in a situation where a fast decision is needed for their critically ill newborn. The clinical research community should promote its use.

## Conclusion

In the SafeBoosC-III trial standard, prior informed consent was most commonly used, deferred consent was used in nearly one-third of participants, while opt-out was only approved in 6 of 61 centres (10%) and only used for 16 out of 1601 participants (1%). This can be considered inappropriate in the context of a trial comparing two commonly used care practices with minimal risks but where parents are usually present with some ability to take in information and make decisions.

### Supplementary Information

**Supplementary Material 1.**

## Data Availability

The datasets will be made available after reasonable request to the corresponding author
