# Supplementary material for: Alternative consent methods used in the multinational, pragmatic, randomised clinical trial SafeBoosC-III
Source: Trials. 2024 Apr 4;25:236. doi: 10.1186/s13063-024-08074-0 (PMC10996265; doi:10.1186/s13063-024-08074-0)
Supplement: Supplementary file 1 — Supplementary Material 1. [file 13063_2024_8074_MOESM1_ESM.docx]

Dear Editors

I have found no appropriate reporting checklist for this research article. If any suggestion, please write me and I’m very sorry for the inconvenience.

Kind regards

Maria Vestager
